# Knowledge about peri-implant diseases in fifth year dental students of the University of Barcelona (Spain): A cross-sectional study

**DOI:** 10.4317/jced.63022

**Published:** 2025-08-01

**Authors:** Anna Terradellas-Luengo, Marta García-García, Rui Figueiredo, Eduard Valmaseda-Castellón, Alba Sánchez-Torres

**Affiliations:** 1 DDS. Faculty of Medicine and Health Sciences, University of Barcelona, Barcelona, Spain; 2 DDS, MS, PhD. IDIBELL Research Institute, Barcelona, Spain

## Abstract

**Background:**

The replacement of missing teeth with implants is a frequent procedure in dental practice, and is usually associated with high implant survival rates. Peri-implant diseases are very common, however. It is therefore essential for clinicians to periodically examine and evaluate implant-supported restorations. The main objective of the present study was to evaluate the level of knowledge about peri-implant diseases and determine the possible associations with demographic variables in fifth year dental students of the University of Barcelona (Spain).

**Material and Methods:**

A cross-sectional study of the knowledge about peri-implant diseases was carried out using a survey administered to the fifth year dental students of the University of Barcelona. Descriptive and bivariate analyses using the Student t-test and Pearson correlation coefficient were made. The significance level was set at *p* < 0.05.

**Results:**

Eighty-three students were included in the study. A mean of 8.7 points (correct answers) were recorded out of a total of 15 possible points. Good student knowledge was observed in relation to prevention and risk factors. No significant associations were observed between the number of correct answers and the different variables studied. Almost all the students (95.2%) underscored the need for further training in peri-implant diseases.

**Conclusions:**

Fifth year students have average level knowledge about peri-implant diseases, with good mastery of prevention and risk factors. There is a general perception that knowledge about prevention and risk factors and the treatment of peri-implant diseases is not enough. In addition, the students are aware of their shortcomings and recognize the need for further training in this field.

** Key words:**Dental education, peri-implant diseases, dental implant, knowledge, dental students, dental education.

## Introduction

The placement of dental implants to replace missing teeth has become increasingly popular, and is generally associated with high implant success rates. Nevertheless, biological complications related to osseointegrated implants have also acquired considerable relevance in dental practice [1-3].

Peri-implant disease can be divided into two distinct disorders. On one hand, peri-implant mucositis is an inflammatory condition of the mucosa surrounding an endosseous implant, without the loss of supporting peri-implant bone [4]. On the other hand, peri-implantitis is a plaque-associated pathological condition occurring in tissues around dental implants and characterized by inflammation of the peri-implant mucosa with subsequent progressive loss of supporting bone [5]. According to Lee *et al*. [6], the worldwide prevalence of peri-implant mucositis and peri-implantitis at patient level is 19.8% and 46.8%, respectively. In Spain, Rodrigo *et al*. [7] found the prevalence of peri-implant mucositis and peri-implantitis at patient level to be as high as 27% and 24%, respectively.

Inflammation is identified by visual inspection of the tissues (swelling and/or erythema), and another key diagnostic tool is peri-implant probing to detect bleeding and/or suppuration, applying a light maximum force of 0.25 N3. Periapical radiographs remain the radiological test of choice for exploring peri-implant bone level, though the technique can only assess the mesial and distal aspects [8].

The presence of biofilm on the surface of the implant is the main etiological factor in the development of peri-implant disease. For this reason, the use of maintenance therapy and mechanical biofilm control should be regarded as the standard treatment for managing mucositis and preventing peri-implantitis [9,10].

Compliance with peri-implant maintenance seems to be crucial for preventing peri-implant diseases. The periodicity of maintenance should be individualized according to the risk factors inherent to each patient, including the history of periodontitis, poor oral hygiene, the severity of the disease, lack of compliance with peri-implant maintenance therapy, and smoking, among others [10-12].

Based on the data found in the literature, an increased susceptibility to peri-implant disease has been found in patients with a history of periodontitis, smoking, a lack of peri-implant maintenance therapy, diabetes mellitus and poor plaque control [12].

The management of peri-implant disease includes mechanical debridement (also known as non-surgical treatment) to remove biofilm and calculus from the implant surface. This is a key procedure for both primary and secondary prevention. However, non-surgical treatment alone is not effective in resolving peri-implantitis, and a surgical approach is needed including the use of resective, regenerative or combined techniques, depending on the morphology of the existing bone defect [13,14]. This in turn must be followed by maintenance therapy at appropriate intervals [15].

The present study was carried out to evaluate the level of knowledge about peri-implant diseases and determine the possible associations between demographic variables and the knowledge of fifth year dental students of the University of Barcelona (Spain). In addition, an analysis was made of student perception of their knowledge and training received in this field.

## Material and Methods

A cross-sectional study was carried out from March to May 2022 in the dental clinic of the University of Barcelona (Spain), involving a survey on the knowledge about peri-implant diseases among fifth year (second semester) dental students. The study was approved by the local Ethics Committee (protocol number 16/2022) and complied with the recommendations of the Declaration of Helsinki on research involving human subjects [16]. The STROBE guidelines for reporting cross-sectional studies were followed [17]. All subjects gave written informed consent before entering the study.

The sample size was calculated using the G*Power version 3.0 application (Heinrich-Heine-Universitat, Dusseldorf, Germany). Assuming an α value of 0.05 and a statistical power of 80% for a finite population of 88 students, a total sample size of 72 subjects was required.

The survey comprised the following sections: i) personal and professional data, measured with a multiple response questionnaire; ii) knowledge about the diagnosis, prevention, risk factors and treatment of peri-implant diseases using a test with four possible answers; iii) three clinical cases with four possible answers; and iv) questions referred to personal perception of knowledge and professional training, using a scale from 0 (null) to 10 (very good) (Supplement 1) (http://www.medicinaoral.com/medoralfree01/aop/jced_63022_s01.pdf). A thorough literature search was made to select relevant and up-to-date scientific literature published since 2016 in PubMed (MEDLINE) using the key words: “peri-implant diseases”, “prevention”, “risk factors” and “treatment” in order to develop the questions of the survey regarding the diagnosis, prevention, risk factors and treatment of peri-implant diseases. In addition, the survey contained three clinical cases that included clinical data (probing depth, presence of bleeding or suppuration), radiological information and an image (clinical or radiological), provided by a specialist in peri-implant diseases (MGG). A pilot version of the survey was tested with 10 students to ensure the accuracy of the construct and to detect questions generating possible doubts.

The passing grade was defined as 7 points, and the highest score was 15. The level of knowledge was grouped into high (12-15 points), average (7-11 points) and low (1-6 points).

The data analysis was carried out using the SPSS® version 27 statistical package (IBM, USA). The normal distribution of the main outcome variable (number of correct answers) was assessed using the Shapiro-Wilk test. Descriptive and bivariate analyses were performed using the Student t-test and Pearson correlation coefficient. The significance level was set at *p* < 0.05.

## Results

A total of 83 fifth year dental students of the University of Barcelona (Spain) participated in the survey. There were 71 females (85.5%) and 12 males (14.5%). Table 1 shows the main characteristics of the students included in the study. Regarding age, 61.5% of the students (*n*=51) were between 21-24 years of age, 32.5% (*n*=27) between 25-30 years of age, and 6% (*n*=5) over 30 years of age. Forty-three students (51.8%) had completed high school before entering university; 36 (43.4%) had completed a vocational training course, specifically Oral Hygiene or Dental Prosthetics; three (3.6%) came from a university degree; and one student (1.2%) took the entrance examination for those over 25 years of age. As to the reason for choosing the degree in Dentistry, most of the students reported vocation 73.5% (*n*=61). Forty-four students (53%) took an elective course related to implantology, while 39 (47%) selected none.

**Figure 1:**
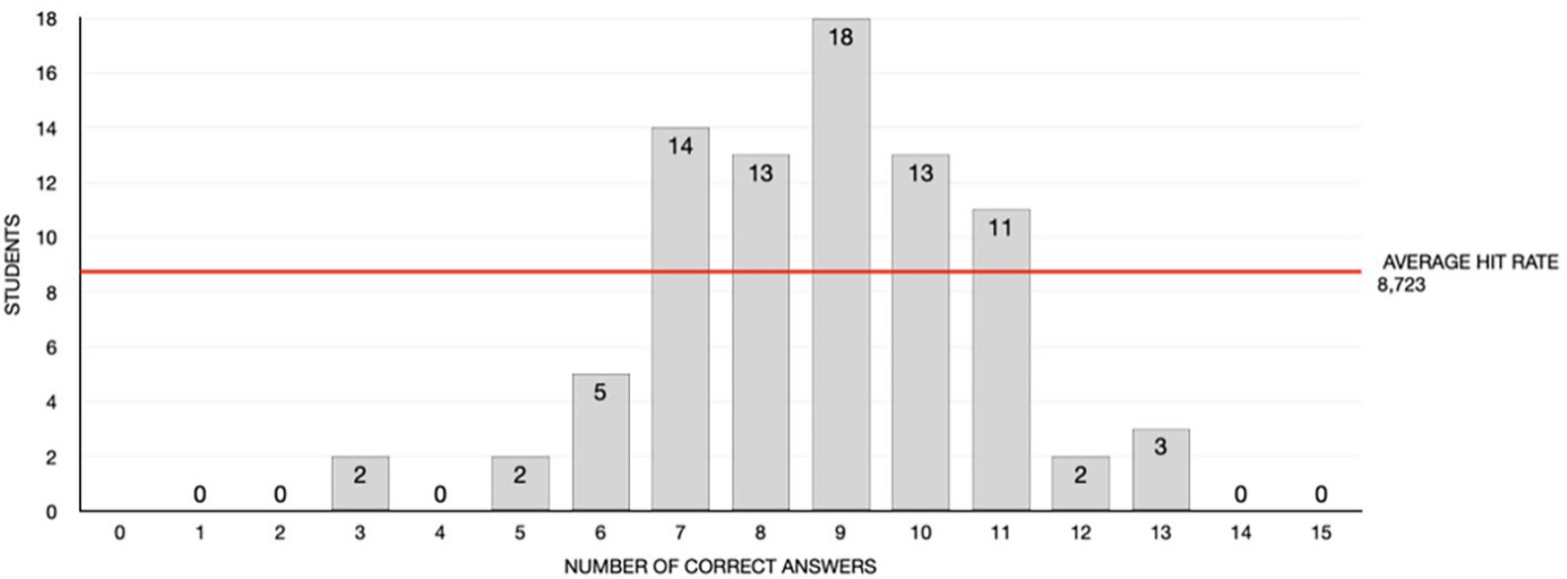
Knowledge scores (number of correct answers) of the students included in the study.

**Figure 2:**
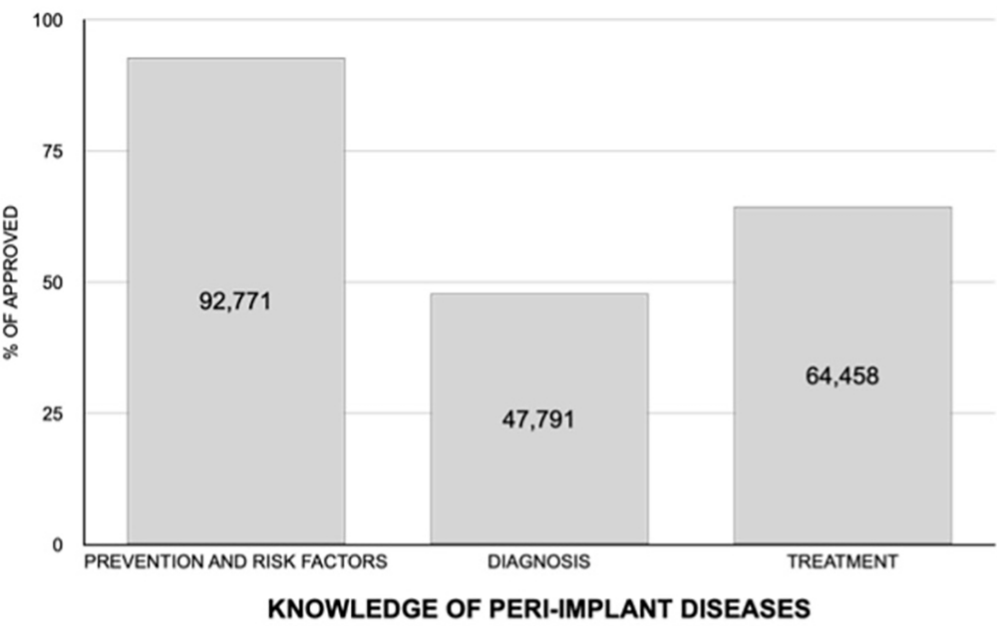
Knowledge of peri-implant diseases of the students included in the study.

In relation to the study variables, the number of correct answers showed a normal distribution (*p*=0.352), with a mean value of 8.7 (standard deviation [SD] = 2). An average level of knowledge (number of correct answers) was recorded in 69 of those surveyed, while 5 of the students showed a high level and the remaining 9 exhibited a low level of knowledge (Fig. 1). The students obtained higher scores on prevention and risk factors, with a percentage of correct answers of 92.8%. In contrast, the questions referred to diagnosis and treatment resulted in a lower percentage of correct answers (47.8% and 64.5%, respectively) (Fig. 2). No differences were observed between demographic variables and the number of correct answers ( Table 1).

No significant association was observed between the self-perception variables and the level of knowledge of the participants in terms of diagnosis (Pearson correlation coefficient, r=0.169; *p*=0.169) and treatment (r=0.664; *p*=0.664), respectively ( Table 2). A positive correlation was recorded in terms of prevention and risk factors, however (r=0.35; *p*=0.010).

Eighty participants (93.4%) considered it advisable to increase training and the theoretical hours dedicated to implant dentistry in pregraduate education. Similarly, 79 of the students (95.2%) expressed the need to receive further training in the subject for their future dental practice.

## Discussion

The main objective of the present study was to evaluate the level of knowledge about peri-implant diseases and determine the possible associations with demographic variables in fifth year dental students of the University of Barcelona (Spain). The results showed 83.1% of the evaluated pregraduate students to have an average level of knowledge of peri-implant diseases, while 10.8% had a low level of knowledge, and only 6.0% exhibited a high level of knowledge. No significant associations were observed between the number of correct answers and the demographic variables of the population.

Student self-assessment of knowledge was evaluated in relation to the diagnosis, prevention and risk factors, and the treatment of peri-implant diseases. No significant association was found between the number of correct answers and student self-assessment of knowledge referred to diagnosis and treatment, though a positive correlation was found in relation to the prevention and risk factors of peri-implant diseases. The students were also questioned about the training received in the field of peri-implant diseases during the university degree. Almost all of the participants considered it advisable to increase training and the theoretical hours dedicated to implant dentistry in pregraduate education, and most of them expressed the need to receive further training in the subject for their future dental practice.

Nowadays, many dental treatments involve the placement of implants. Consequently, as future dental professionals, students need to have some knowledge about implants and peri-implant diseases in order to ensure good diagnosis, prevention and treatment for securing implant success. Our results show that 83.1% of the pregraduate students had an average level of knowledge about peri-implant diseases. While this may be regarded as acceptable, the students should have better knowledge of this subject, which is relevant to many pregraduate subjects such as surgery, periodontics or preventive dentistry.

Our findings coincide with those of a previous study by Madi *et al*. [18] who reported an intermediate to low level of knowledge about peri-implant diseases and conditions. These authors analyzed the level of knowledge referred to the etiology, diagnosis and management of peri-implantitis in senior undergraduate dental students at Imam Abdulrahman Bin Faisal University in Saudi Arabia. They used an online survey with questions addressing the characteristics of the participants; the etiology, diagnosis and treatment of peri-implantitis; and the use of antibiotics in peri-implant diseases. Most of the participants were seen to have a good knowledge of the diagnosis (87.6% were aware of the inflammatory condition of peri-implantitis; 77.9% recognized bacteria as playing an etiological role) and risk factors (82.0% identified smoking and 80.5% identified periodontitis as risk factors). However, the students had less knowledge about the diagnosis and treatment of peri-implantitis (only 50.2% considered bleeding on probing with a probing depth of 6 mm and bone loss at consecutive visits to require surgical treatment of inflammation around implants). These findings are in line with our own results, where the students had fewer correct answers in relation to the diagnosis and treatment of peri-implantitis.

As has been mentioned above, the surveyed undergraduate students showed greater knowledge about the prevention and risk factors of peri-implant diseases than about the diagnosis and treatment of these disorders, which could be a problem for future dental practice. An interesting observation on comparing the number of correct answers and student self-assessment in the different fields is that the participants had the impression of not knowing enough about the prevention and risk factors of peri-implant diseases, despite the fact that this was the area in which they obtained the best scores. Since the success of peri-implantitis treatment is known to be conditioned by initial bone loss, an early diagnosis is crucial [19]. New dentists therefore must be familiarized with the diagnostic aspects of these disorders. Regarding treatment, the limited knowledge observed is understandable, since treatment is usually addressed in Master’s degrees or specialized training in Oral Surgery and Periodontology. However, modern dental practice is multidisciplinary, and it would be desirable for students during the degree to learn how to perform the most basic treatments, such as non-surgical management. The large number of patients who have implants supports the importance of training in implantology during the dental degree, so that the future dentists can offer comprehensive quality care to their patients [20]. Dentists should know the indications and contraindications for the placement of implants. In this context, all dentists should be able to diagnose and prevent disorders associated with dental implants, such as peri-implant diseases.

The knowledge referred to peri-implant diseases acquired by the students is exclusively provided in the context of the dental degree, with the dedication of a few theoretical hours in subjects such as Oral Surgery or Periodontology, with no specific practices for observing or treating such patients. It is curious that no notable changes were observed in the results of those participants who had enrolled in some optional subjects in the area of implantology. This is probably because the students are only familiarized with the disease, without acquiring any real skills in this field.

Another interesting observation is the importance given by the students to the need for redefining training in implantology, with a greater emphasis on peri-implant diseases during the degree, in view of their relevance in current dentistry. Almost all the students considered further training in this area to be advisable. These results are in line with those published by Sánchez-Garcés *et al*. [21] involving undergraduate students in the third and fourth year of the dental degree in the University of Barcelona. The authors used a survey with questions addressing basic knowledge in implantology; perception of the training received; and ways in which the students would like to receive training. The results showed that the students considered themselves to be poorly informed about implant dentistry, and they expressed a wish to receive more information during undergraduate training.

Until relatively recently, peri-implant diseases were only addressed in the context of Master’s degrees or specialized training in Oral Surgery and Periodontology. As mentioned at the First European Consensus on University Education in Implantology 20 in Prague in 2008, treatment with implants is increasingly part of routine rehabilitation procedures. The dental graduate competencies in implantology included in the consensus comprise knowledge and understanding of the diseases that affect the peri-implant tissues, identifying their etiology, pathogenesis, progression and risk factors; skills in diagnosing and treating advanced peri-implant lesions; and awareness of the treatment options for these diseases. Undergraduate dental curricula therefore should include training in proper procedures for correct diagnosis, risk assessment and maintenance procedures to ensure implant success. Those skills not acquired during the dental degree will have to be acquired in postgraduate programs. In this regard, such programs should be made available in order to offer quality and up-to-date patient care.

A limitation of the present study is its low validity, since it is an observational study based on a survey. Another limitation is that it only includes students. Future research should compare opinions between students and opinion leaders in universities regarding the importance of training in skills related to the diagnosis and treatment of peri-implant diseases, which are areas where students seem to have greater difficulties.

According to our knowledge, the present study is the first to analyze knowledge about peri-implant diseases in final year dental students in Spanish Universities. It would be advisable to compare the results obtained with those of other studies carried out in Spanish and European universities, and to evaluate and determine University teaching levels in implantology and peri-implant diseases. As a cornerstone of dental pedagogy, it is essential to insist on the importance of exhaustive and proficient instruction combining theoretical information, clinical application and interpersonal skills at the same level.

## Conclusions

Final year dental students were seen to have moderate knowledge about peri-implant diseases. Good knowledge referred to disease prevention and risk factors was recorded, though knowledge about the diagnosis and treatment of peri-implant diseases was limited. There were no significant differences in the number of correct answers associated with student age, gender, previous studies including higher education, elective courses taken, preferred specialty or reason for studying dentistry. The students considered themselves to have insufficient knowledge about the prevention and risk factors of peri-implant diseases. In contrast, discordant results were observed between student perceived knowledge about diagnosis and treatment and actual knowledge as evidenced by the study survey. Further training in peri-implant diseases is needed to complete the knowledge of undergraduates in dentistry.

## Figures and Tables

**Table 1:** Demographic data of the participants in the study. N (%).

Demographic data of the participants in the study. N (%)			Number of correct answers
			P-value
Gender	Male	12 (14.5%)	0.682
	Female	71 (85.4%)	
Age (years)	21-24	51 (61.5%)	0.929
	25-29	27 (32.5%)	
	>30	5 (6.0%)	
Previous studies	High school diploma	43 (51.8%)	0.422
	Advanced vocational training course	36 (43.4%)	
	University degree	3 (3.6%)	
	University entrance exam for people over 25 years of age	1 (1.2%)	
Elective course related to implantology	Yes	44 (53.0%)	0.983
	No	39 (47.0%)	
Reason for choosing the degree	Vocation	61 (73.5%)	0.617
	Not achieving the grade needed for another degree	14 (16.9%)	
	Recommendation by dentist relative	5 (6.0%)	
	Recommendation by friend	3 (3.6%)	

† t-test; ‡ one-way ANOVA

**Table 2:** Association between participant self-perception and knowledge in the different areas (Pearson correlation test).

Association between participant self-perception and knowledge in the different areas (Pearson correlation test).		
Diagnosis	Pearson correlation	0.152
	Significance	0.169
Prevention and risk factors	Person correlation	0.353
	Significance	0.010*
Treatment	Pearson correlation	-0.048
	Significance	0.664

## Data Availability

The datasets used and/or analyzed during the current study are available from the corresponding author.
